# Comparative Strength Analysis of Helical Gears with Different Tooth Profiles Using FEM

**DOI:** 10.3390/ma19194078

**Published:** 2026-09-23

**Authors:** Paweł Fudali, Jacek Pacana

**Affiliations:** Faculty of Mechanical Engineering and Aeronautics, Rzeszow University of Technology, al. Powstancow Warszawy 12, 35-029 Rzeszow, Poland; pfudali@prz.edu.pl

**Keywords:** gears, gearboxes, FEM, CAD systems, Novikov profile, tooth outline

## Abstract

This paper presents a strength analysis of helical gears for several selected tooth profiles. Four analogous gear design solutions with identical geometry and functional features were compared, with differences limited solely to the parameters of the gear rim. The primary focus of the research was on gears featuring convex–concave profiles based on Novikov and sinusoidal tooth profiles, which were compared against the standard involute profile. Solid models of the investigated gears were prepared using a CAD (Computer-Aided Design) method developed by the authors. The modelling process, which replicates the generating gear cutting method, allowed for highly accurate gear models to be obtained. The computational models of circular-arc tooth profile gears prepared in this manner were subsequently used for numerical FEM (Finite Element Method) analyses in ANSYS 2025 R2 software. As part of the numerical computations, instantaneous contact patterns and total contact patterns for the loaded gear drive were determined. Furthermore, the stress state at the tooth root and the surface stresses on the contacting tooth flanks were specified. Stress plots in these critical areas, which are essential for gear strength, were generated alongside distribution maps of regions prone to failure. Finally, an evaluation and comparative analysis of the results obtained for all considered gear models were conducted. The conclusions derived from the study provide insights into the stress distribution within critical regions of gears with different circular-arc tooth profiles.

## 1. Introduction

Involute gears remain by far the most widely used type in industrial machinery. Apart from the well-known advantages and disadvantages of this convex profile, it should be noted that, given the current state of knowledge, the potential for improving their functionality has practically been exhausted. Therefore, designers are increasingly turning to alternative solutions—either entirely new or previously known and described, yet hitherto impossible to implement widely due to the lack of sufficient technological or material capabilities. A solution that could replace imperfect involute profiles is gears with convex–concave profiles. These solutions, inspired by nature, have already been described multiple times; however, there was a lack of means to accurately verify their strength, as well as the technological capabilities required for their widespread adoption. Recent advances in computer techniques, along with manufacturing capabilities utilising CNC (Computer Numerical Control) machines and additive manufacturing methods, provide a foundation for revisiting detailed investigations and assessments of at least selected convex–concave gearings.

Gears of this type were first patented by R.H. Smith in 1912 [1,2], but a detailed description is lacking in the literature. On the other hand, the first convex–concave gear system was the VBB (Vickers-Bostock-Bramley) herringbone gear [1,2], patented in 1922 by F.J. Bostock and C. Bramley-Moore from the Vickers company (Sheffield, England). This gear initially operated successfully, but tooth breakage occurred after some time. The failure to resolve the problem of increasing its durability led to the abandonment of research into this gear system [1,3].

Subsequent designs of gears with convex–concave tooth profiles were also presented by Walker [2,3], Wolski [4], Hlebanja [5,6], Sendyka [3,7], and Buczek [8,9]. However, the concepts of Wildhaber [1,10,11] and Novikov [2,12,13] have aroused the greatest interest to date. Wildhaber patented his solution in 1926. It was characterised by a circular-arc profile in the section normal to the tooth line, and identical curvatures of the tooth flanks of the mating gears. A development of this concept was the M. L. Novikov gear [12], patented in 1955. It differed from the Wildhaber gear [10] in that the circular-arc profile occurs in the transverse section. A more important difference, however, concerns the different radii for the concave tooth profile surfaces of the mating gears in Novikov’s profiles. Thanks to this modification, the gear is less sensitive to changes in the centre distance caused by manufacturing errors of its components [2,14]. However, because Wildhaber-Novikov gearing is low-contact and satisfies the law of constant gearing ratio for only a single point of contact, they must be used exclusively as helical gears.

Despite their many advantages, they are not widely used, which may be attributed to the necessity of using special tools, in contrast to the universal tooling applicable to the involute profile.

Prior literature reviews indicate that Novikov gears transmit 1.5 to 1.7 times greater loads [9,15,16,17] than helical gears with an involute tooth profile possessing the same geometrical and material properties, which ought to be verified using modern computational tools. During the peak period of research into Novikov tooth profiles, technical and computational capabilities were significantly more limited than those available today. Analytical calculations relied primarily on complex geometric and kinematic analyses, while the implementation of selected designs was preceded by lengthy experimental testing on prototypes. Consequently, a comprehensive assessment of the stress state across the entire gear model—as well as the observation of variations in specific gear regions at different meshing stages—was impossible. Such capabilities have only been unlocked by modern computational software systems, which substantially broaden the design possibilities for modern engineers. Nevertheless, the majority of literature on this somewhat overlooked profile still relies on analytical calculations, contact geometry analysis (including the use of solid models), and experimental studies [18,19,20]. Consequently, regarding Novikov gear drives, there is a lack of literature addressing FEM-based numerical analysis and attempting to evaluate the scope of their application, particularly in comparison with the conventional involute profile.

In addition to Novikov gears, which possess non-standard tooth profiles, other gears with convex–concave profiles also arouse significant interest among designers. An example of an alternative solution to involute gears is the sinusoidal gear.

Currently developed solutions are based on E. Wildhaber’s patent from over half a century ago [21]. Research results published in the works of numerous authors [22,23,24,25,26] confirm the better load-carrying capacity of the sinusoidal gear, its higher bending strength due to a wider tooth root compared to involute gears, the occurrence of lower friction on the mating surfaces, and lower contact stresses in the mesh, which also contributes to a lower noise level [27,28,29,30]. Currently, research results for many modified sinusoidal profiles are being presented, but their common feature is a characteristic convex–concave profile defined in the normal section [31,32,33].

This profile, representing a trigonometric sine function, should be symmetrical. Profiles of this type find application in gears with heightened requirements, including planetary gears [34], or non-standard solutions such as strain wave gears (harmonic drives) [35,36,37], as well as in gear pumps [38,39].

The primary objective of this paper is to conduct a comparative stress analysis for various tooth profiles under identical loading conditions, including gears with Novikov profiles, in order to evaluate the viability of their application in comparison with other established solutions. The primary objective of this article is to determine the actual strength of gears with Novikov profiles against the background of other known and widely used solutions. This would enable their broader application in machine drives and perhaps render them an alternative to conventional involute gears, at least in selected types of designs. The most frequent requirement imposed on alternative gear types is a higher load-carrying capacity and durability compared to traditional ones. General knowledge suggests that to meet this requirement, gears with convex–concave tooth profiles should be applied.

In order to verify the validity of the hypotheses presented above regarding gears with circular-arc tooth profiles, a comparative analysis of selected design solutions was carried out using FEM.

## 2. Materials and Methods

This paper presents an analysis of the relationships between the tooth profile and gear strength for four selected tooth profiles. The conducted investigations included three cylindrical helical gears featuring fundamental convex–concave tooth profiles, and a standard involute gear system was adopted to verify the obtained results. The decisive factor in choosing these specific profiles is that, for each of them, both mating gears can be manufactured using a single set of tools, which can be a critical consideration for their industrial application. Two Novikov profiles, a sinusoidal profile, and—as a reference for them—an involute profile with a module of m = 2.5 mm were selected for the analysis. This work presents two selected variants of the Novikov profile that exhibit meshing with two lines of contact. The first type is a gear system with a Novikov profile based on the basic rack profile, the parameters of which were presented in the GOST 15023 standard from 1969 [40]. The second Novikov gear system possesses a tooth profile according to the GOST 30224 standard from 1996 [41], which is proposed for gears made of materials with a hardness of greater than 35 HRC. To simplify the notation, these profiles will be referred to hereinafter according to the year in which they were standardised, namely as “Novikov-69” and “Novikov-96”. All these gear systems were designed by adopting the same input parameters regarding loads, using identical materials, and for identical operating conditions. Based on the standards [40,41,42,43] and the literature [1,2,44], a gear system was designed to transmit a power of 16 kW and operate at a nominal speed of 1200 rpm. The basic rack profile for the Novikov-69 profile is shown in Figure 1, and the values of the parameters dependent on the module are given in Table 1.

Figure 2 presents the modified basic rack profile for the Novikov-96 gear system based on GOST 30224-96. Table 2 lists the parameters of this profile extracted from the specified standard, which are recommended for modules smaller than 3.15 mm.

It should be noted that the parameters of the Novikov profile specified in the GOST 30224 standard [41] from 1996 contain contradictions that prevented the correct modelling of the tool profile. These errors did not significantly affect the shape of the profile, nor should they cause any changes in the gear strength or engagement characteristics. However, they were identified, and the recommended method for correcting the geometry of Novikov-96 gears was presented in a previous publication by the authors [45].

The next system under investigation is a sinusoidal gear, in which the tooth profile features a curvilinear outline that represents a trigonometric function. A sinusoidal basic rack profile described by a sine function [25,28] was adopted, which is symmetrical with respect to the reference line. The sinusoidal profile occurs exclusively in the normal plane of the tooth; therefore, such solutions should more generally be termed sinusoid-derived gears. However, for the sake of simplicity, the term sinusoidal gear will be used in the analysed case, which should be directly related to the profile of the applied tool.

Figure 3 illustrates the sinusoidal basic rack profile adopted in the study. In this case, the crest of the sine wave forms the root of the tooth space.

A standard profile, which was a conventional involute profile, was adopted to verify the investigated convex–concave profiles. This is a well-known and widely used tooth shape that has been described multiple times in the literature [1,16,17]. For the comparative analysis, a basic rack profile was adopted in which the tooth thickness on the reference line is equal to the width of the tooth space. The involute basic rack profile used for modelling the gears and performing numerical calculations is shown in Figure 4. The basic parameters of the involute profile with a normal module value of m_n_ = 2.5 mm were adopted on the basis of the literature and the PN-ISO 53:2001 standard [42,43,46].

Analytical calculations were carried out for all analysed variants to determine their geometrical parameters resulting from strength requirements. Subsequently, for the presented profiles, the tool outlines were generated in AutoCAD 2026, maintaining the same basic geometrical and functional parameters—such as the normal module, face width, gear ratio, and transmitted power—across all analysed gear systems.

In order to obtain solid models of all the gears for the analysed systems, a subtractive manufacturing simulation process was prepared and conducted within CAD software [46,47]. The resulting models featured non-smooth surfaces, which led to difficulties and limitations during the phase of defining the FEM calculations. Therefore, the tooth flanks of all gears were additionally smoothed using surfaces fitted through their key profile points [48]. This procedure enabled the maintenance of uniform finite element mesh parameters in the numerical calculations for all analysed cases. The solid models used in the FEM analysis ensured high computational accuracy, replicated the actual gear machining methods with exceptional detail, and maintained consistency with the analytically determined tooth profile [48,49].

## 3. Preparation of Numerical Calculations

Numerical FEM analysis regarding the strength and determination of meshing conditions for helical gears is a highly complex problem. Conducting the calculations correctly requires proper preparation of the solid models, appropriate definition and execution of the numerical analysis, and, most importantly, correct verification and evaluation of the obtained results. To achieve sufficiently accurate solutions, high-performance computers and dedicated software must be utilised. Furthermore, extensive knowledge and experience are essential, not only in software operation and FEM theory but also in the field of gear design, strength of materials, and gear operation.

The numerical FEM analysis was carried out using the MECHANICAL module within the ANSYS software suite. Due to the high complexity of the models and the very large memory requirements imposed on the computing server, the numerical calculations incorporated only fragments of the gears, comprising seven teeth for each gear (Figure 5). Such models allow for a correct simulation of the meshing of the five central pairs of teeth and do not affect the quality of the obtained results. Numerical constraints were introduced at the cut-off locations of the models to ensure the required stiffness and displacement of the mesh nodes. This model reduction enables more effective utilisation of hardware capabilities, and in this specific case, allows for a significant increase in the finite element mesh density within the critical regions of the models.

For each of the analysed tooth profiles, the same methods and model definition parameters were applied during the calculation preparation process. For all models imported into ANSYS, a geometrical check was carried out, and the gear models were positioned in accordance with the analytically calculated geometrical parameters specific to a given gear system. Geometrical constraints and loads were introduced, and material parameters were defined. Subsequently, the discretisation process was conducted by meshing the model using hexahedral finite elements. On the mating tooth flanks, the finite element mesh was significantly refined (“A” in Figure 6), which enabled high-quality results regarding contact stresses to be obtained. Each of the computational models contained 1 million finite elements. The calculations involved simulating the meshing of the gears through a pinion rotation of 60°. This range was divided into 1000 steps, thereby allowing for highly accurate observation and interpretation of the results at various stages of engagement for each pair of gears. To determine the instantaneous contact areas and contact patterns on the tooth flanks, frictional contact with a coefficient of friction of 0.1 was defined. The Augmented Lagrange formulation was utilised. A constant friction coefficient of µ = 0.1, representative of mixed lubrication conditions in industrial gearboxes, was assigned to the contact interface [50,51]. The contact detection was assigned to Gauss integration points to ensure smooth stress transition across the contact line.

To reflect the operating conditions of the analysed gear drive in ANSYS, appropriate boundary conditions and loads were defined. A torque of 159 Nm was applied to the inner surface of the gear bore, representing the load acting on the evaluated transmission component. Conversely, a rotational displacement of 60° was assigned to the inner surface of the pinion bore. Adopting this loading scheme enabled forced relative rotation of the gear pair components and facilitated the analysis of their interaction within the meshing zone. Furthermore, to replicate the actual support conditions and eliminate undesirable model displacements, axial translation of both the gear and the pinion was constrained. At the same time, rotation about their respective axes was permitted in accordance with the specified loading conditions. The boundary conditions established in this manner allowed for the reproduction of basic gear operating conditions and enabled the analysis of transient contact conditions across several consecutive teeth.

The preparation of the analysis for each of the four models with differing tooth profiles proceeded in the identical manner. Due to the high finite element mesh density adopted, the computation time was extensive; however, in all cases, the calculations completed without errors, and the expected results were obtained.

Due to the high accuracy expected for the results concerning the flanks of the mating teeth, special attention was paid to ensuring the identical conditions for all analysed models. To determine the most appropriate mesh density for these particularly critical regions, multiple verification calculations were conducted, and the convergence of the obtained solutions was established. The mesh was considered converged when the change in stress values between successive iterations was less than 3%. For all considered profiles, the same number of finite elements across the tooth height and face width, the same type of finite elements, and identical contact conditions were defined.

## 4. Results

The FEM calculation results obtained for all computational variants were subjected to an initial correctness evaluation and compared with one another. The primary format for presenting the solutions is the contour plot of the von Mises equivalent stress distribution on the model surfaces. Exemplary results for the analysed gear systems featuring all four investigated tooth profiles are shown in Figure 7. These results are presented on the models of the driven gears and correspond to the same stage of tooth engagement.

The presented stress distributions reveal distinct variations depending on the different tooth profiles applied. In the case of the sinusoidal and the baseline involute profiles, the stresses are distributed across the entire face width of the gears and are observed on up to three consecutive teeth. For gears with Novikov profiles, engagement occurs on only two consecutive teeth; however, on each individual tooth, two stress regions appear simultaneously in the vicinity of the tooth root and the addendum. This confirms the previously described characteristic of Novikov profiles, where engagement is featured by double-line tooth contact. Furthermore, in Novikov gear systems, the stresses are localised over a smaller surface area rather than across the entire face width of the gears, unlike in the other two cases. Therefore, the maximum von Mises equivalent stress values recorded for the Novikov gears are higher than those for the remaining two profiles. The lowest equivalent stresses—approximately twofold lower—were observed for the gears with the sinusoidal profile. Comparably low stress values on the tooth flanks were recorded for the gears with the involute profile; however, in this case, a localised stress concentration occurred at the tooth edges. Correcting the profile and eliminating the unfavourable engagement of the tooth tips would significantly reduce the stress levels and increase the strength and durability of the gear system.

In order to investigate the loading of individual teeth more thoroughly, plots of stress variations over time were prepared. In a static analysis, time does not represent physical time in terms of dynamics, as inertial forces, accelerations, and damping are omitted. Instead, it serves merely as a control parameter governing the simulation progress. In the first step of the analysis t = 0–1 s, the torque increases linearly until it reaches its full magnitude, whereas in the second step t = 1–2 s, the rotation of the gear drive is performed. The focus was placed on verifying the bending stress values at the tooth roots, which constitutes one of the critical conditions used to determine tooth strength according to the standard [52]. For all analysed gear systems, plots were generated to present the equivalent stress values at points located at the mid-face width of the gears at the tooth roots, designated as “c” in Figure 7a.

The equivalent stress values at the tooth roots, corresponding to all meshing phases, are presented in Figure 8, Figure 9, Figure 10 and Figure 11.

The presented plots clearly illustrate the characteristic loading pattern of each tooth. The shapes of the curves corresponding to the subsequent investigated profiles differ from one another. Furthermore, the maximum stress values at the tooth root are different in each case. The highest values, exceeding 200 MPa, occurred for the Novikov-69 and sinusoidal profiles during the initial phase of engagement. The next lowest root bending stress values were featured by the Novikov-96 profile. These averaged approximately 175 MPa, yet they were still significantly higher than in the case of the reference involute profile. In all cases, the highest root stresses occurred when the gear tooth entered into mesh. At this instant, for each consecutive pair of teeth, contact occurs between the tooth tip of the gear and the root of the pinion. In the subsequent phase of engagement, such high tooth loading no longer occurs in any of the investigated cases. It should also be noted that for the Novikov profiles, the curves also exhibit an extremum in the second phase of engagement. This is because these profiles possess two lines of contact; thus, the second extremum chronologically corresponds to the engagement at the gear tooth root. Compared to the initial phase of engagement, the root bending stresses in this region have lower values because the tooth load acts at a smaller radius. The distinct character of the tooth loading can be clearly observed by comparing the plots obtained for the different tooth profiles with one another. Figure 12 presents the root bending stress plots corresponding to one full meshing cycle. The moment of the first contact between the flanks of the mating teeth was adopted as the common initial point of the plot for all profiles.

As can be seen, the duration of the phases and the entire meshing cycle varies for the different investigated tooth profiles. The presented results provide clear information regarding the unfavourably high stress levels at the tooth root in the case of gear systems with Novikov profiles, as well as a twofold higher frequency of its loading. This may lead to accelerated wear, adverse vibrations, and noise, as well as a greater risk of fatigue failure.

Based on the same models, it is also possible to determine the instantaneous gear contact patterns within the ANSYS software. Thanks to the information acquired in this manner, changes in the position of the contact area of the meshing teeth can be observed dynamically. Unlike tooth contact analyses performed in CAD software, the results obtained using the FEM method provide significantly more accurate data [53,54]. It is possible to observe not only the location of the instantaneous contact patterns but also their deformations and positional shifts resulting from changes in load values. For all investigated tooth profile variants, the results concerning the instantaneous contact patterns on a selected tooth of the gear model are presented (red colour on the Figure 13 and Figure 14). In each case, the results correspond to the instant when the contact area on the selected tooth was at its largest.

The results for the investigated tooth profiles are compared with one another in Figure 13, revealing distinct differences between them. For the Novikov-69 and Novikov-96 profiles, two contact patterns are clearly visible in the vicinity of the tooth addendum and root. Their extent is significantly smaller than in the case of the sinusoidal and involute profiles, which are distributed uniformly across the entire face width of the gears. Furthermore, both the shape of the contact pattern and its surface area are substantially smaller for the Novikov profiles, suggesting the occurrence of higher contact stress values in these regions.

Based on the conducted calculations, it is also possible to determine the areas where mutual contact between the gears occurs during the entire meshing cycle. The total contact patterns were generated within the ANSYS software as the sum of all instantaneous contact patterns for a single gear tooth. The results obtained for all investigated tooth profiles are presented in Figure 14.

The total contact patterns for the sinusoidal and involute profiles cover practically the entire tooth flank. These engagement results were obtained because the basic theoretical profile was adopted for the analysis. Neither transverse tooth profile correction nor longitudinal tooth shape modification along its line was performed. An undesirable outcome is the observed contact pattern also at the gear tooth tip. This may lead to a local stress increase, resulting in chipping of the tooth corner. Such a situation does not occur in the case of the Novikov profiles, as in these instances, the total contact pattern is clearly arranged in two lines without any contact with the tooth tip. The obtained results very clearly confirm the occurrence of double-line contact in convex–concave Novikov gear systems.

The presented contour plot of the contact area distribution on the tooth flanks provides information regarding the correctness of engagement, the possibilities for editing profile parameters, or the predicted wear regions. To acquire precise data regarding loading and strength, the exact stress values at key locations on the tooth flanks must be determined.

Therefore, the locations of these characteristic regions were established, and the contact stress values were recorded at specific points. For the sinusoidal and involute gears, a characteristic point located on the pitch diameter and simultaneously at the mid-face width of the gear was designated. This point was marked on the flank of an exemplary tooth and labelled with the symbol “D” in Figure 14d. In the case of both gear systems featuring the Novikov profile, two characteristic points were designated on the tooth flanks, labelled as “H” and “F” in Figure 14a. These points were located at the mid-face width of the gears at heights determined by the tooth contact lines.

Since the conducted FEM calculations involved a simulation of continuous engagement of multiple pairs of teeth, it was possible to generate contact stress plots at the specified points based on the recorded data. The plots prepared in this manner for all analysed gear systems with differing tooth profiles are presented in Figure 15, Figure 16, Figure 17 and Figure 18. Each colour of the plot corresponds to the engagement of consecutive pairs of gear teeth.

The obtained plots are characterised by a sudden, local increase in contact stress values at the moment of contact occurrence, and a lack of their significant influence once the instantaneous contact pattern moves beyond the measurement point. Furthermore, in the case of the Novikov-69 and Novikov-96 profiles, two contact stress concentrations occurred for each tooth, phase-shifted in the engagement cycle corresponding to the two lines of contact. The contact stress values recorded at the characteristic points varied significantly depending on the applied tooth profile. The highest contact stress values occurred for the convex–concave profiles (Figure 14a,b) and exceeded 1000 MPa. Given the gear material adopted in the calculations, which was a case-hardening alloy steel, the allowable stress values were not exceeded. For the selected chromium-molybdenum steel and an assumed load cycle count of 10^6^ cycles, the allowable Hertzian contact stress is 1362 MPa. However, this condition should be noted and conclusions drawn, as significantly lower contact stress levels were obtained for the remaining two profiles. Additionally, in the case of the sinusoidal profile, the maximum contact stress values were below 380 MPa and were simultaneously lower than those occurring for the involute profile.

## 5. Summary and Conclusions

The conducted strength analysis of gear systems for selected circular-arc profiles enabled a qualitative evaluation of the analysed solutions and the formulation of preliminary conclusions. Currently, the industry is dominated by involute gears, which is primarily due to the well-known and proven universal method of their manufacturing. However, as is well-known, they exhibit unfavourable engagement resulting from continuous sliding friction between the mating tooth flanks. To avoid this phenomenon, teeth with circular-arc profiles that roll over one another without sliding can be utilised in gear systems [9,12,16]. This paper compared the strength of various tooth profiles to determine whether the benefits resulting from superior engagement conditions allow for the practical industrial application of Novikov gear systems.

A definitive answer as to which of the investigated gear systems is the best overall was not obtained. To quantitatively evaluate the results obtained for each of the analysed solutions, they are summarised in Table 3. For sinusoidal and involute tooth profiles exhibiting a single contact trace on the flank, the average values are identical to the maximum values. In the case of Novikov profiles, the average values were calculated as the arithmetic mean of the maximum stress values from the two contact traces occurring during single-pair mesh. In Figure 15 and Figure 16, these two traces corresponding to a single tooth flank are denoted by a common colour. 

Among the analysed circular-arc profiles, the lowest equivalent stress values were achieved for the Novikov-96 profile. This profile exhibits over 25% lower root bending stress values than those of the other convex–concave profiles. This indicates that the modifications introduced in subsequent versions of the Novikov profile are favourable in nature.

The evaluation of the distribution and magnitude of contact stresses on the mating tooth flanks does not yield a positive assessment for the Novikov gear systems. In comparison, both the sinusoidal profile and the reference involute profile perform better. Due to the characteristic engagement along two contact lines inherent to Novikov profiles, their contact patterns are restricted solely to these specific regions. Conversely, for the sinusoidal profile, the instantaneous contact area is distributed over a larger surface, and the total contact pattern covers almost the entire flank of the mating teeth. Consequently, the resulting contact stress values for the Novikov profiles are definitively the highest among all the investigated gear systems. However, based on the preliminary evaluation of the solutions presented, it is not possible to draw definitive conclusions regarding a decisive superiority of any single solution over the others. A more detailed analysis is required to assess the effect of local geometry on stress concentration in the tooth contact zone and to identify the critical regions for structural strength [55].

In none of the analysed cases were the allowable stress values exceeded, which means each of these solutions can be successfully applied. Bearing in mind the advantages resulting from the engagement of circular-arc tooth profiles, it is worthwhile to undertake further challenges aimed at improving their strength. Future work should focus on altering selected parameters of the Novikov profile to reduce the stress values within the immediate tooth engagement area. This might be achievable by correcting the radii values that define the basic profile, which will constitute the next stage of the authors’ research. The proposal of new, even highly complex tooth profiles no longer poses a significant challenge given the availability of computer techniques in engineering design. Furthermore, manufacturing capabilities have advanced substantially since the time the Novikov profile was originally developed; therefore, there are high hopes that subsequent research will yield numerous solutions satisfying the ever-growing strength and durability expectations for gear systems, including those with circular-arc tooth profiles.

## Figures and Tables

**Figure 1:**
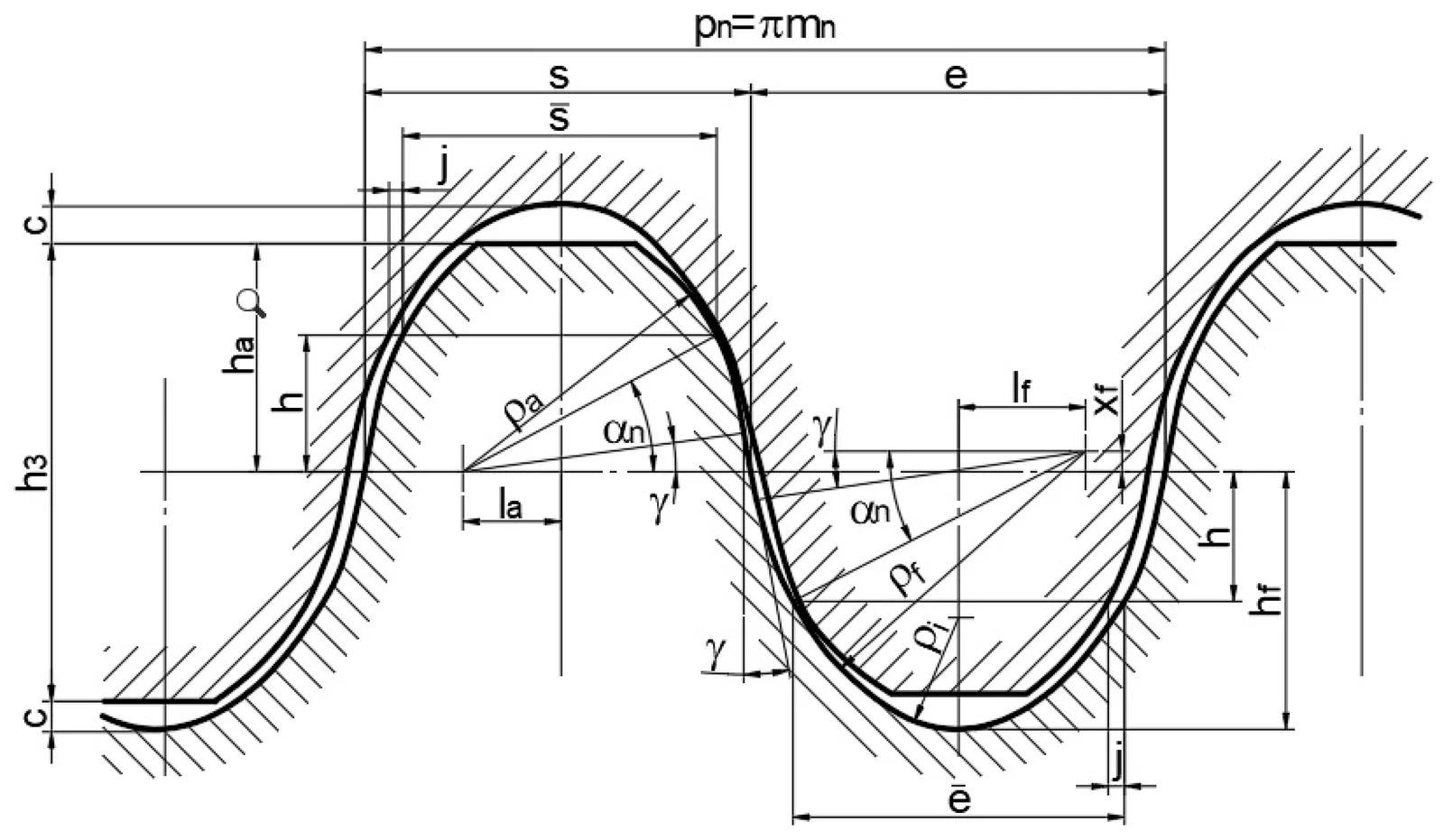
Basic rack profile for a Novikov gear system with two lines of mesh based on GOST 15023-69 [40]: ρ_a_—radius of the arc forming the convex section of the tooth profile (tooth tip); ρ_f_—radius of the arc forming the concave section of the tooth profile (tooth root); ρ_i_—fillet radius at the tooth root base; α_n_—pressure angle; γ—profile angle of the rectilinear connection of the arcs forming the convex and concave sections of the tooth profile.

**Figure 2:**
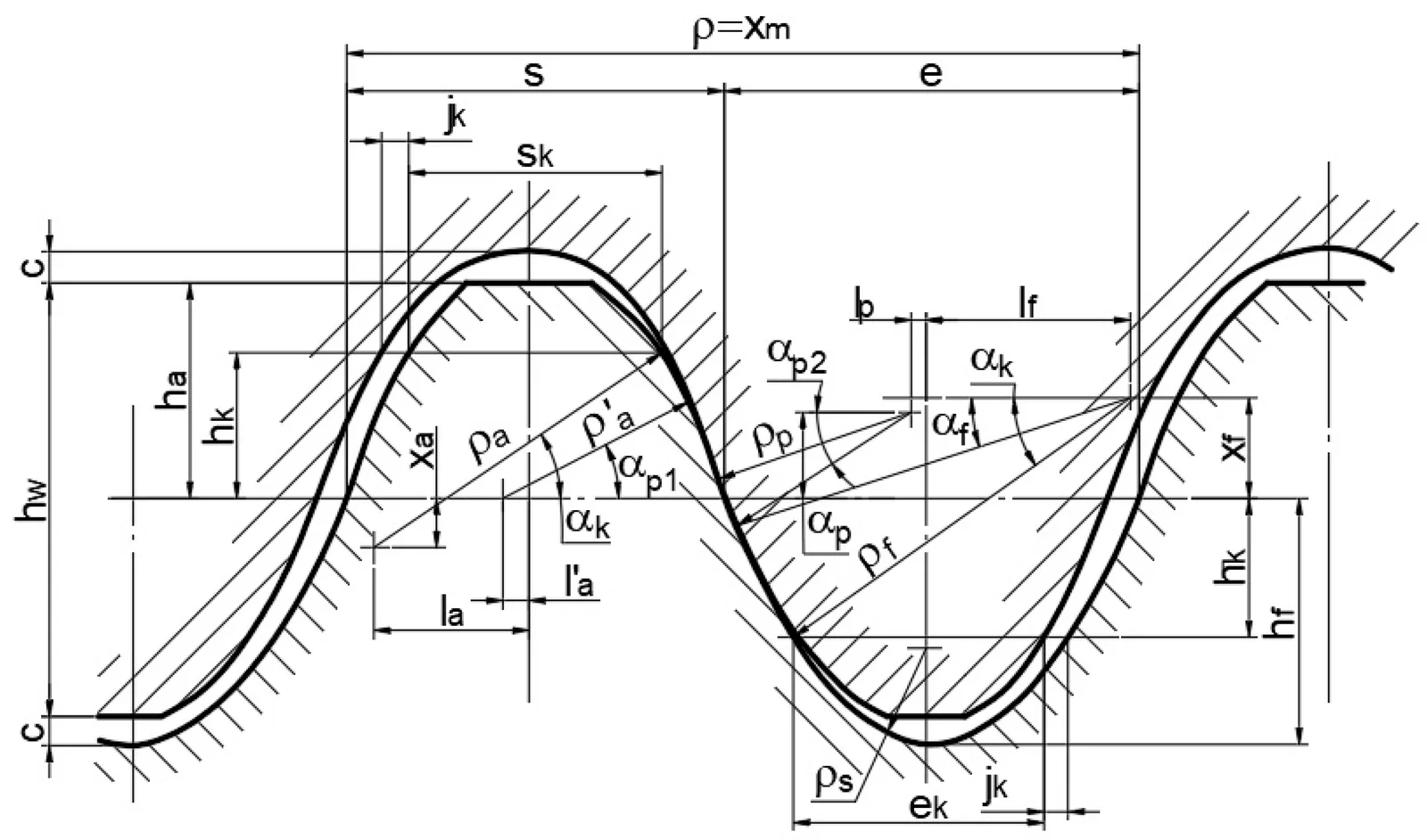
Basic rack profile for a modified Novikov gear system with two lines of mesh based on GOST 30224-96 [41]: ρ_a_—radius of curvature of the active tip profile; ρ_f_—radius of curvature of the active root profile; ρ′_a_—radius of curvature of the convex section of the peripolar transition curve; ρ_p_—radius of curvature of the concave section of the peripolar transition curve; α_p1_—profile angle at the point of tangency of the circles of radii ρ_a_ and ρ′_a_; α_p2_—profile angle at the point of tangency of the circles of radii ρ′_a_ and ρ_p_; α_f_—profile angle at the intersection point of the circles of radii ρ_p_ and ρ_f_; c—radial clearance; α_k_—profile angle at the contact point.

**Figure 3:**
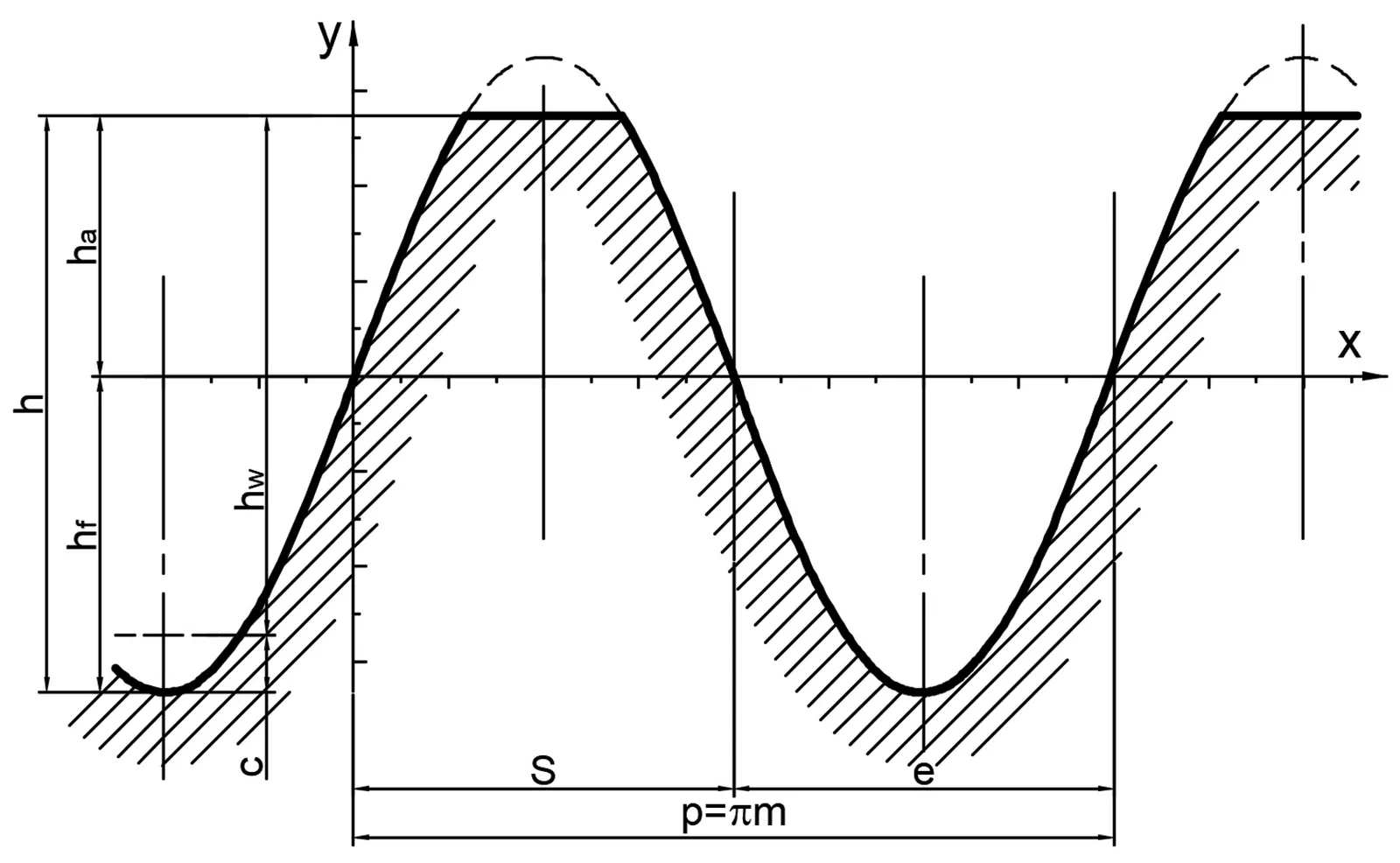
Cutting edge profile of a rack cutter for machining sinusoid-derived gears [45] with basic parameters: h_a_ = 0.9, h_f_ = 1.15, c = 0.25.

**Figure 4:**
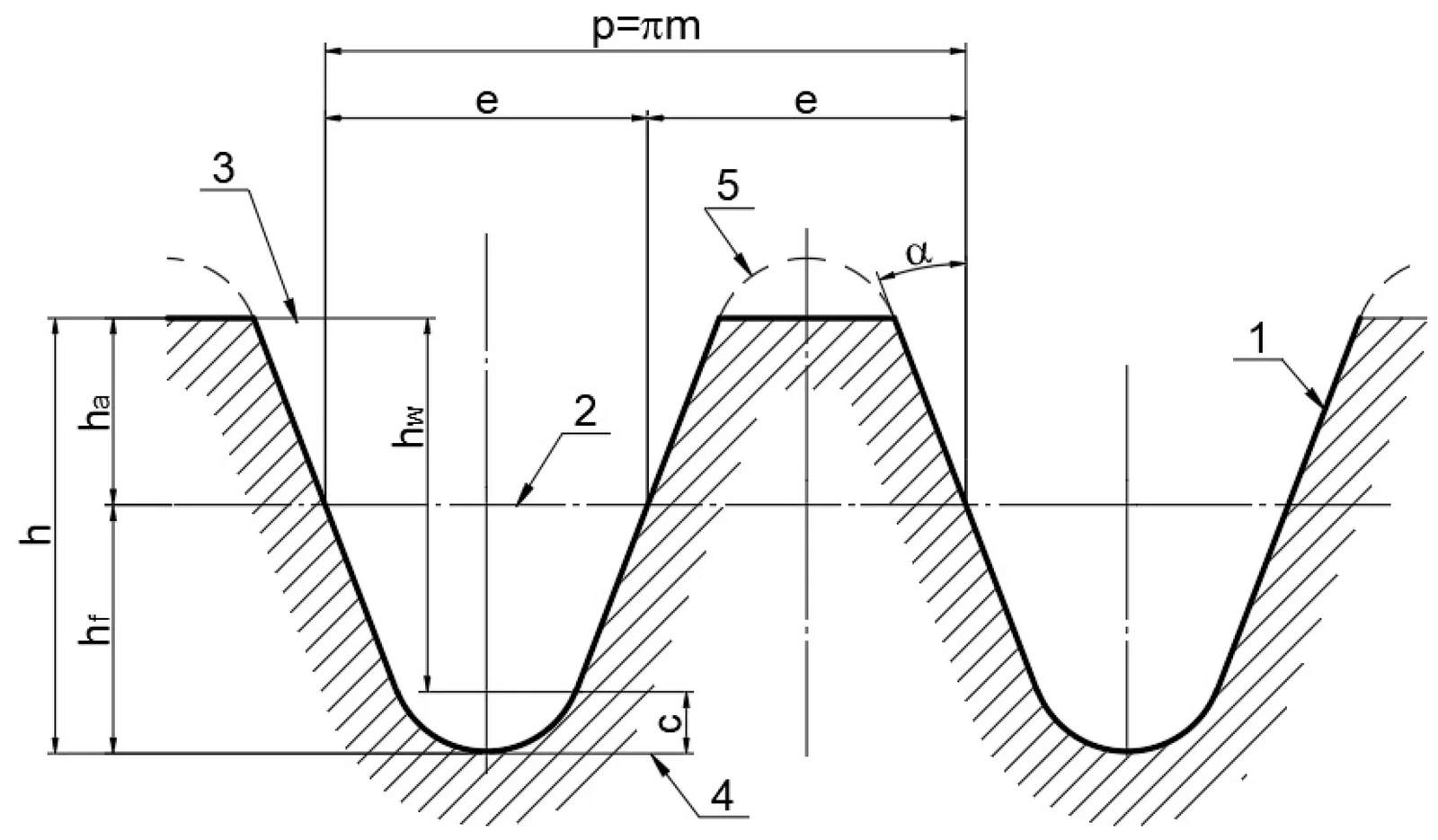
Involute basic rack profile and conjugate profile: 1, basic rack profile; 2, reference line; 3, addendum line (crest line); 4, dedendum line (root line); 5, conjugate basic rack profile [46], h_a_ = 0.9, h_f_ = 1.15, c = 0.25.

**Figure 5:**
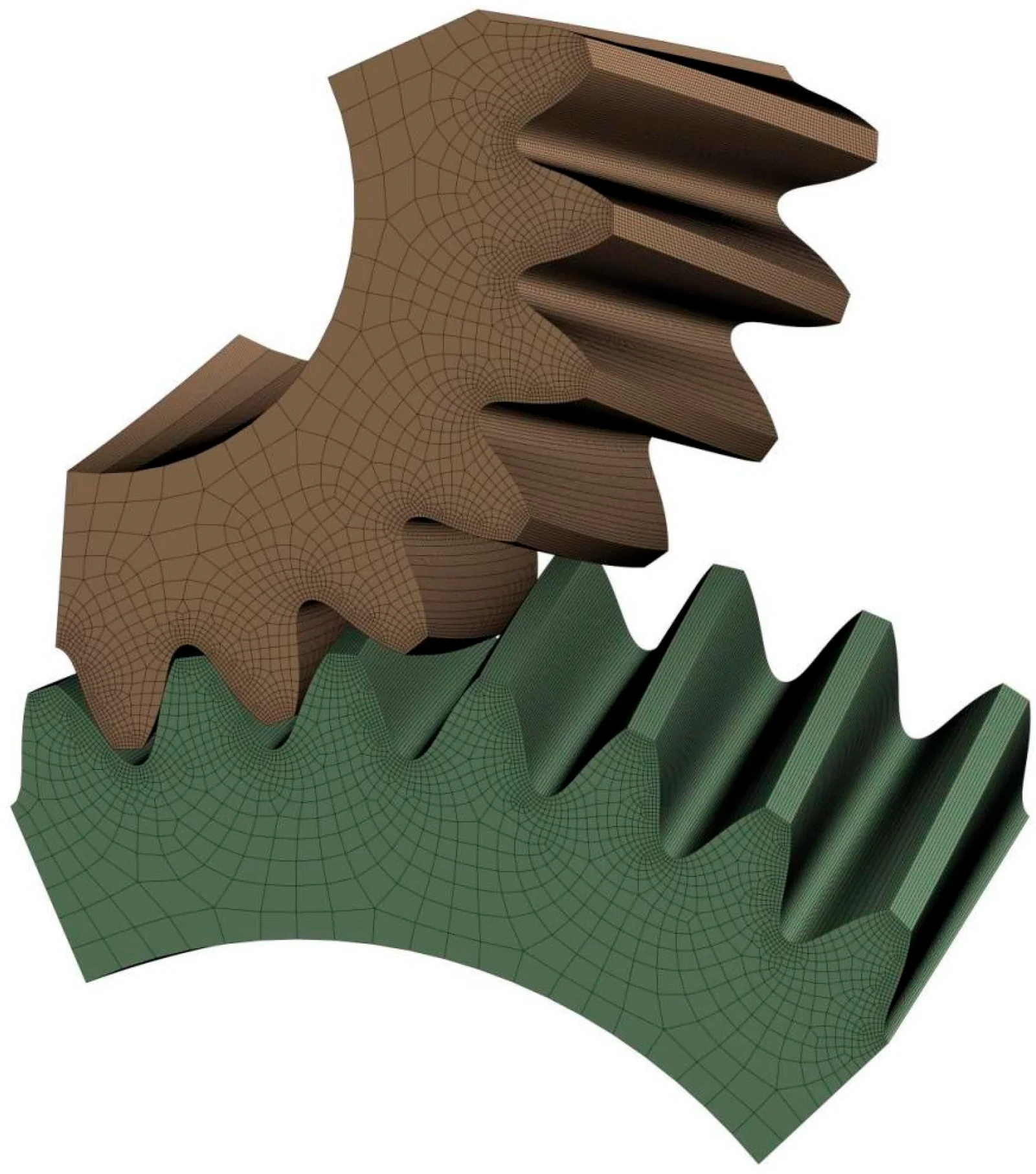
General view of the sinusoidal gear models within the ANSYS preprocessor following discretization.

**Figure 6:**
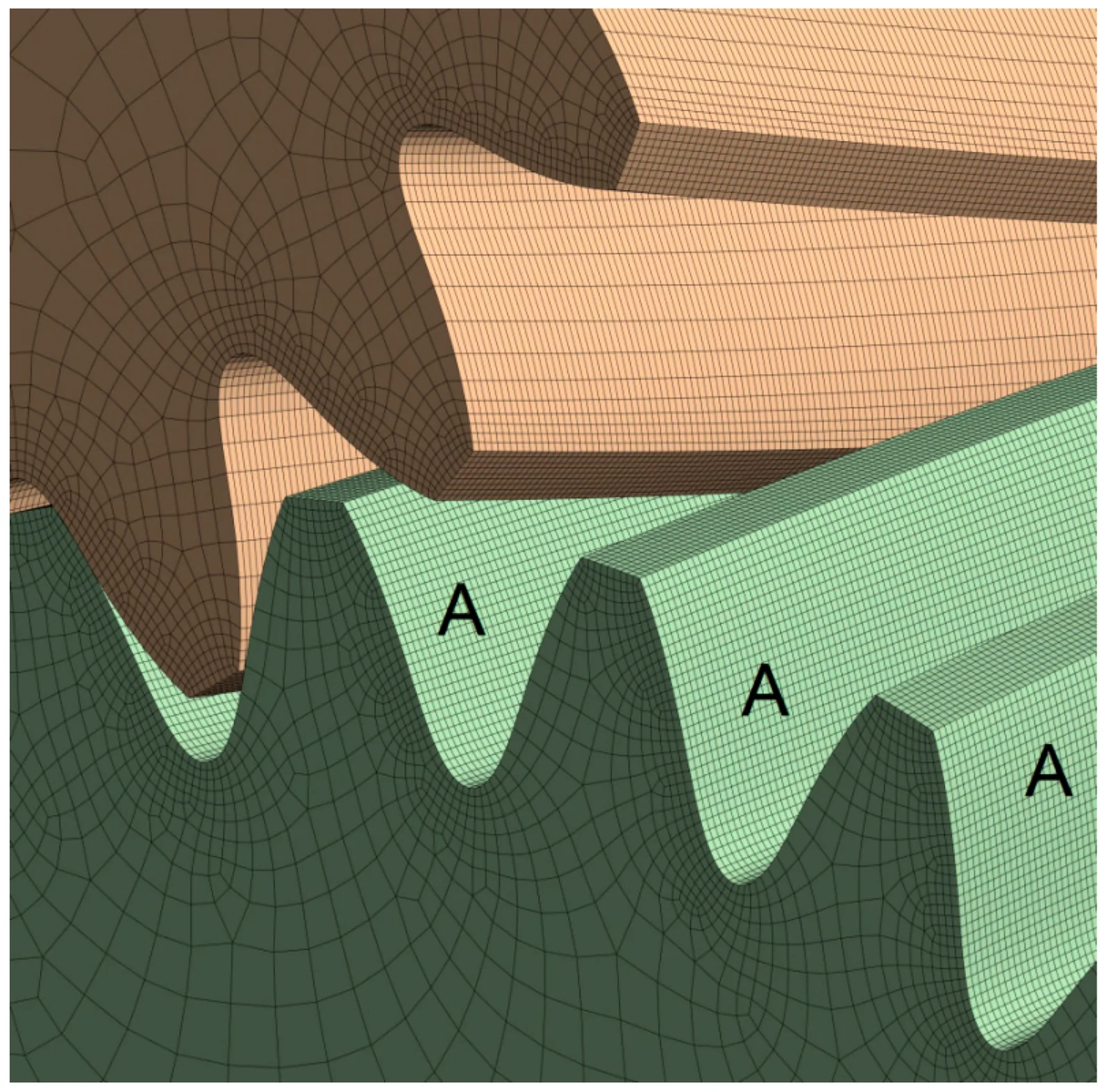
View of the tooth flanks with visible finite element mesh refinement on the active flank (A).

**Figure 7:**
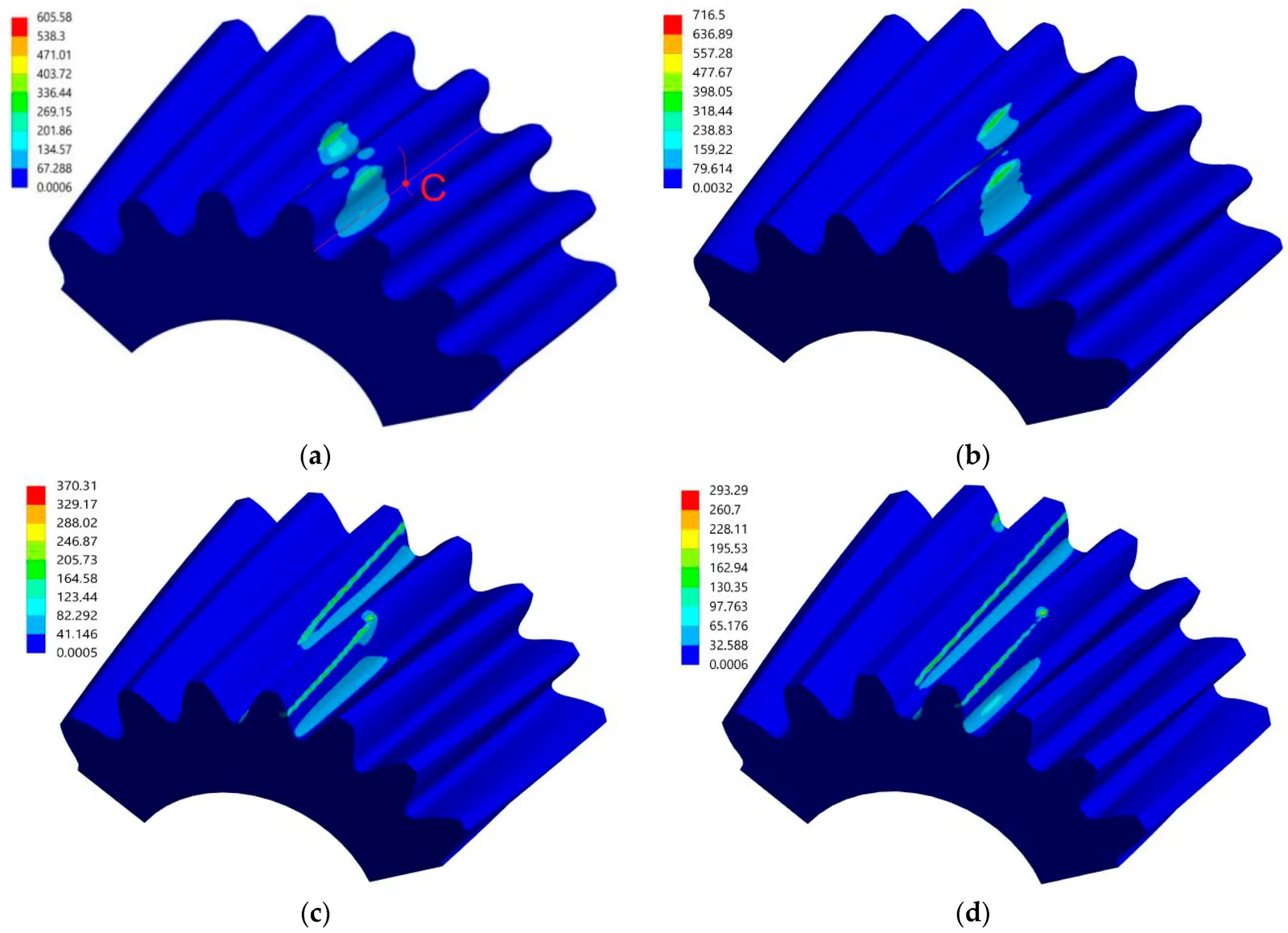
Distribution of von Mises equivalent stresses on the models of driven gears for gear systems with the following profiles: (**a**) Novikov-69, (**b**) Novikov-96, (**c**) sinusoid-derived, (**d**) involute.

**Figure 8:**
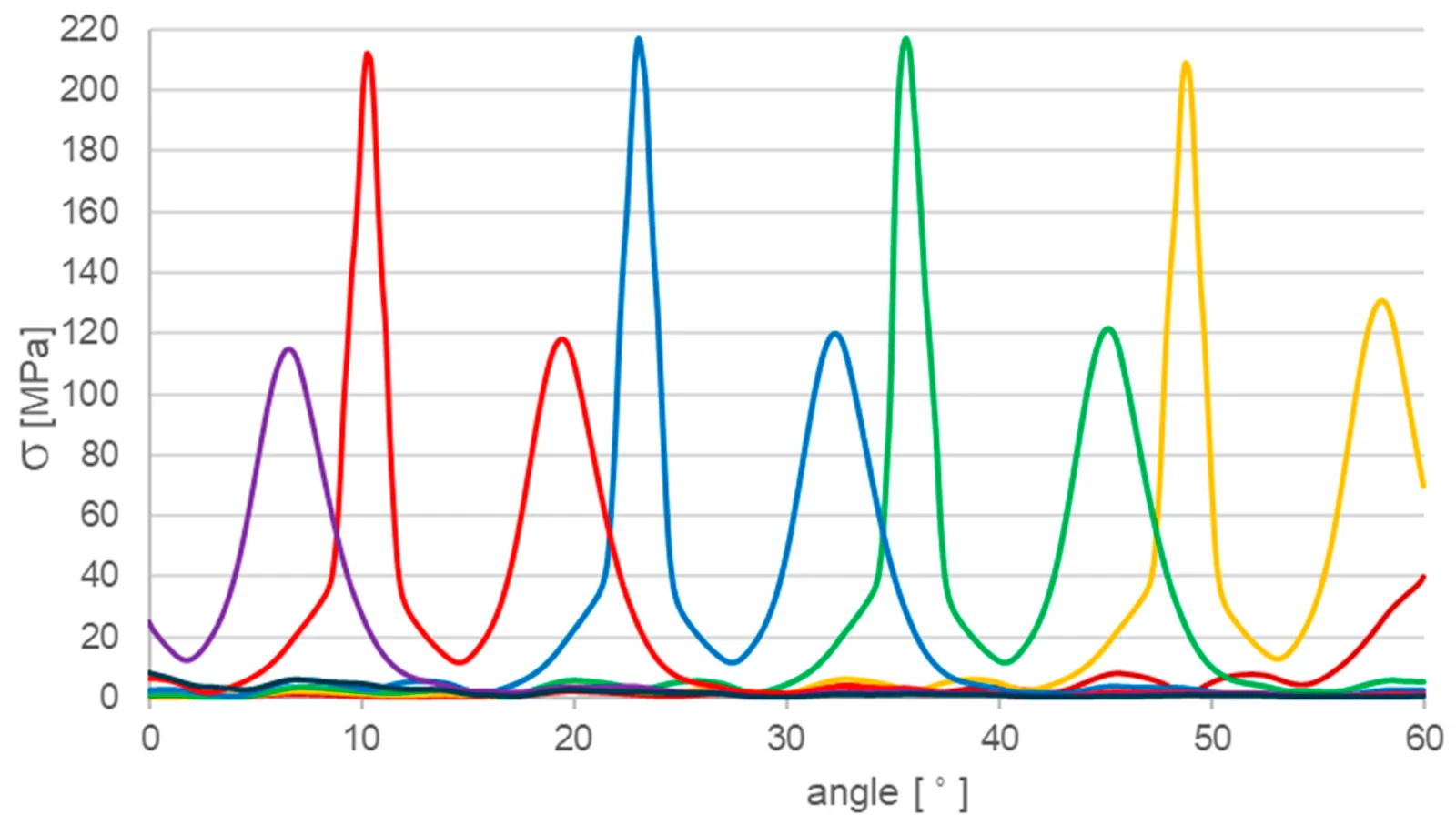
Plot of von Mises equivalent stresses at the tooth root for the Novikov-69 gear model as a function of the pinion rotation angle.

**Figure 9:**
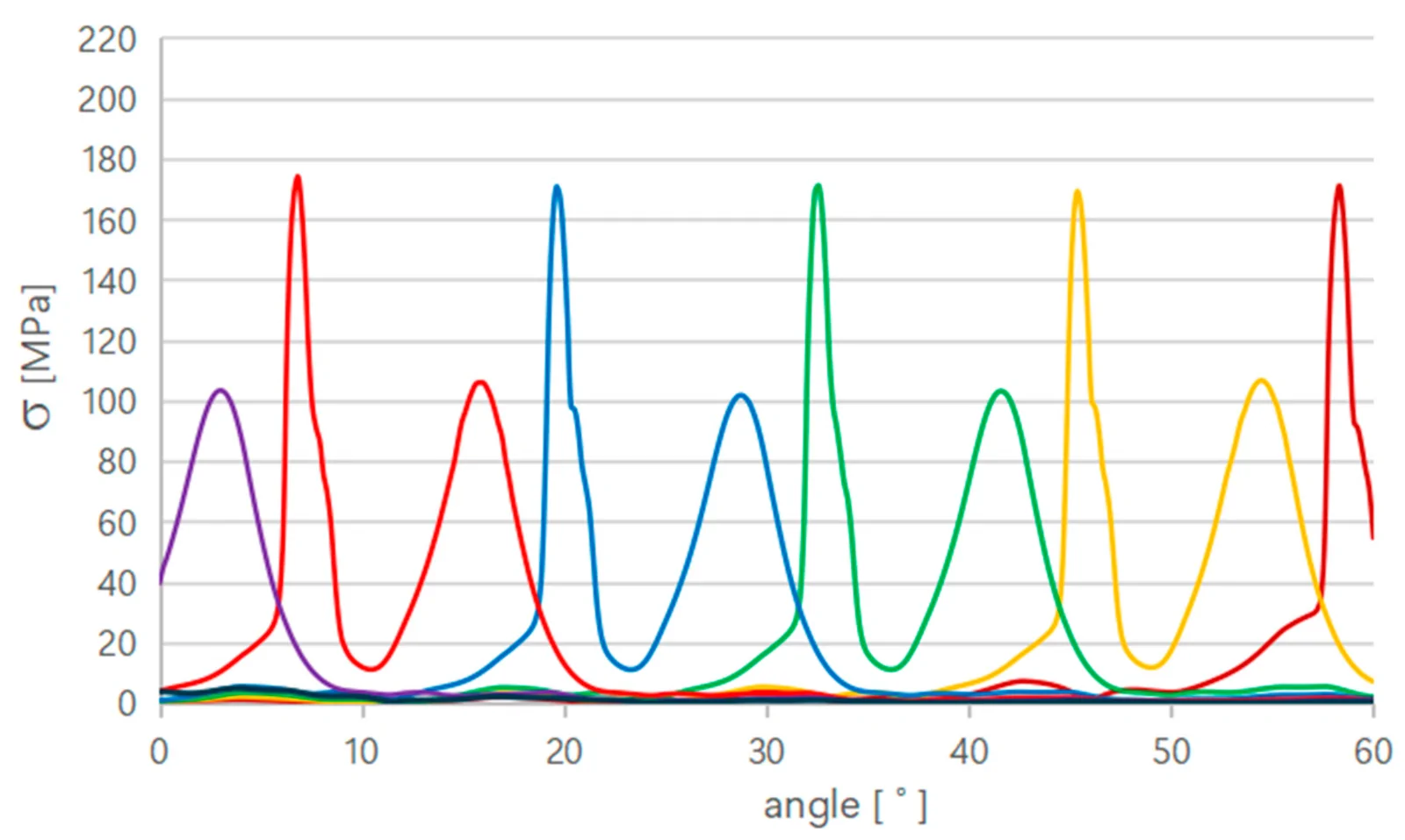
Plot of von Mises equivalent stresses at the tooth root for the Novikov-96 gear model as a function of the pinion rotation angle.

**Figure 10:**
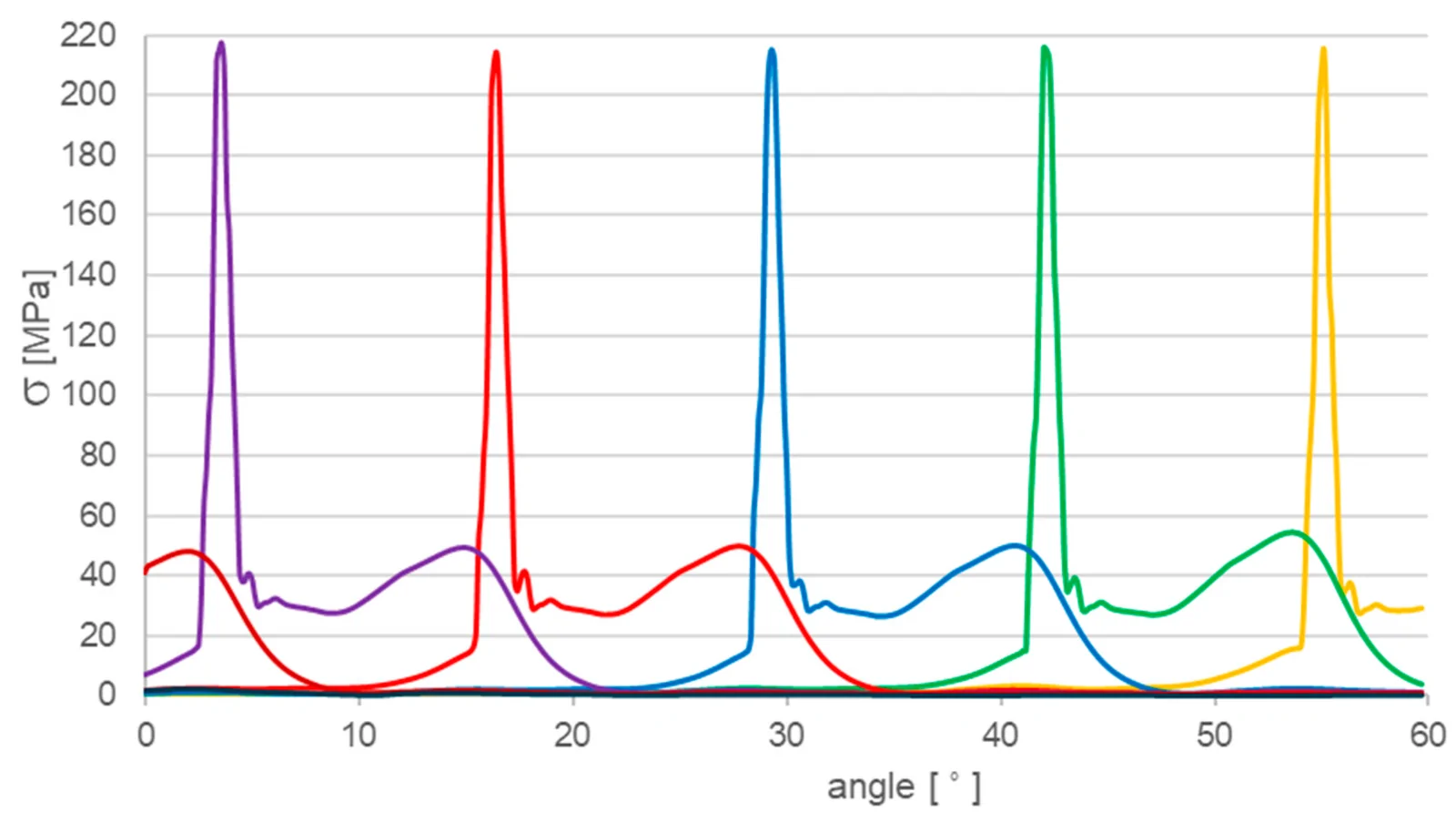
Plot of von Mises equivalent stresses at the tooth root for the sinusoidal gear model as a function of the pinion rotation angle.

**Figure 11:**
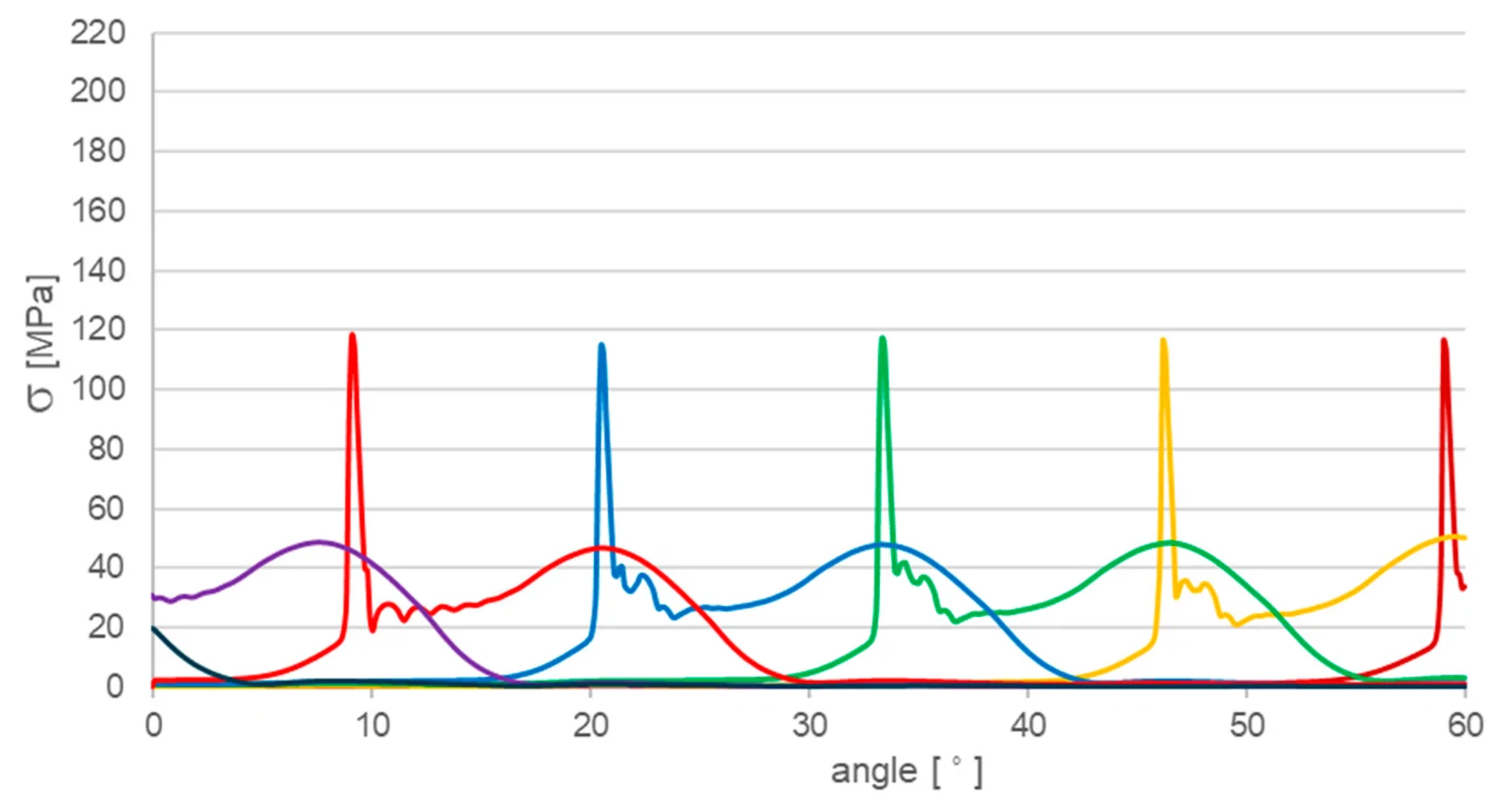
Plot of von Mises equivalent stresses at the tooth root for the involute gear model as a function of the pinion rotation angle.

**Figure 12:**
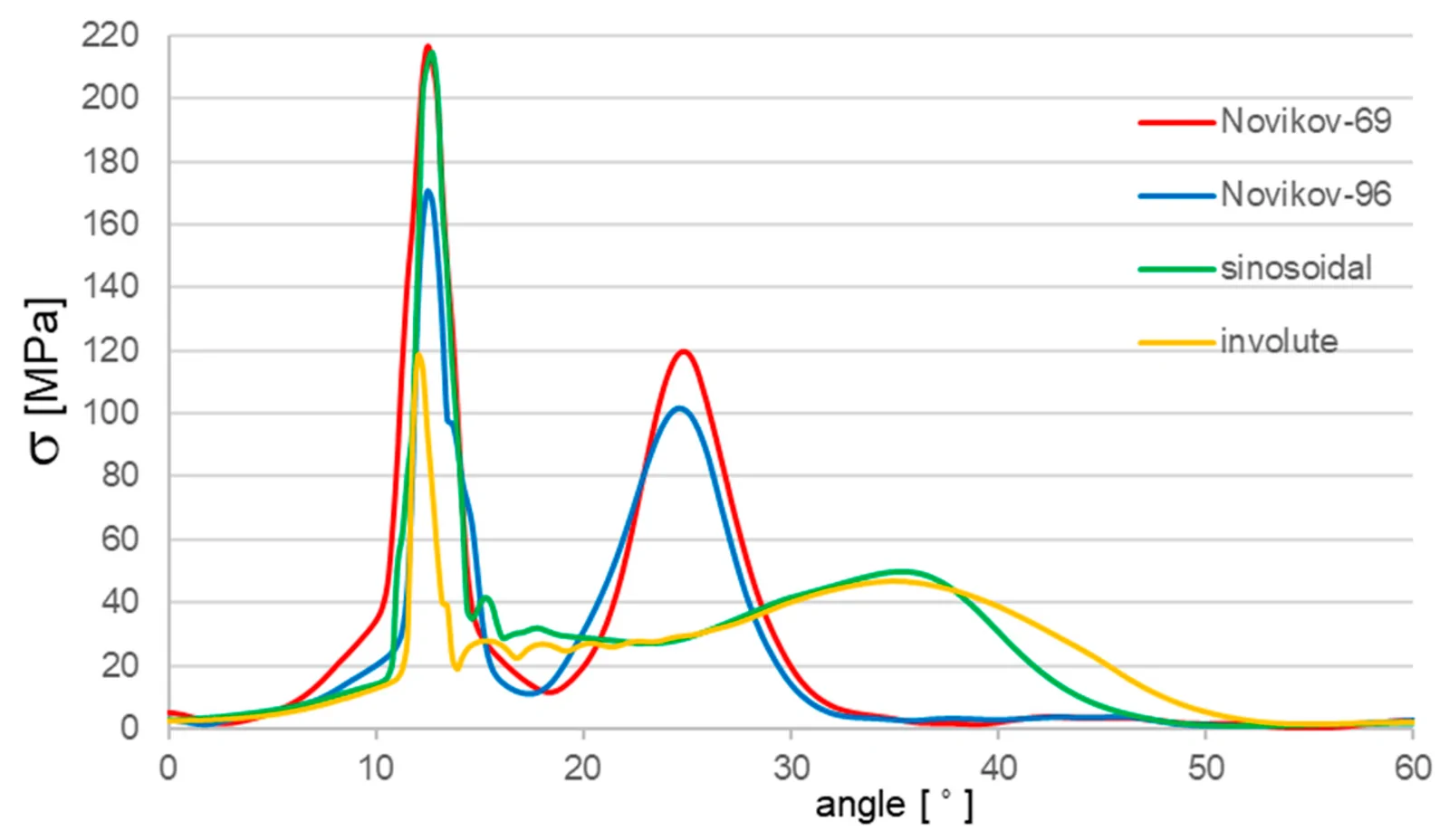
Comparison of root bending stress plots for teeth with the investigated profiles as a function of the pinion rotation angle.

**Figure 13:**
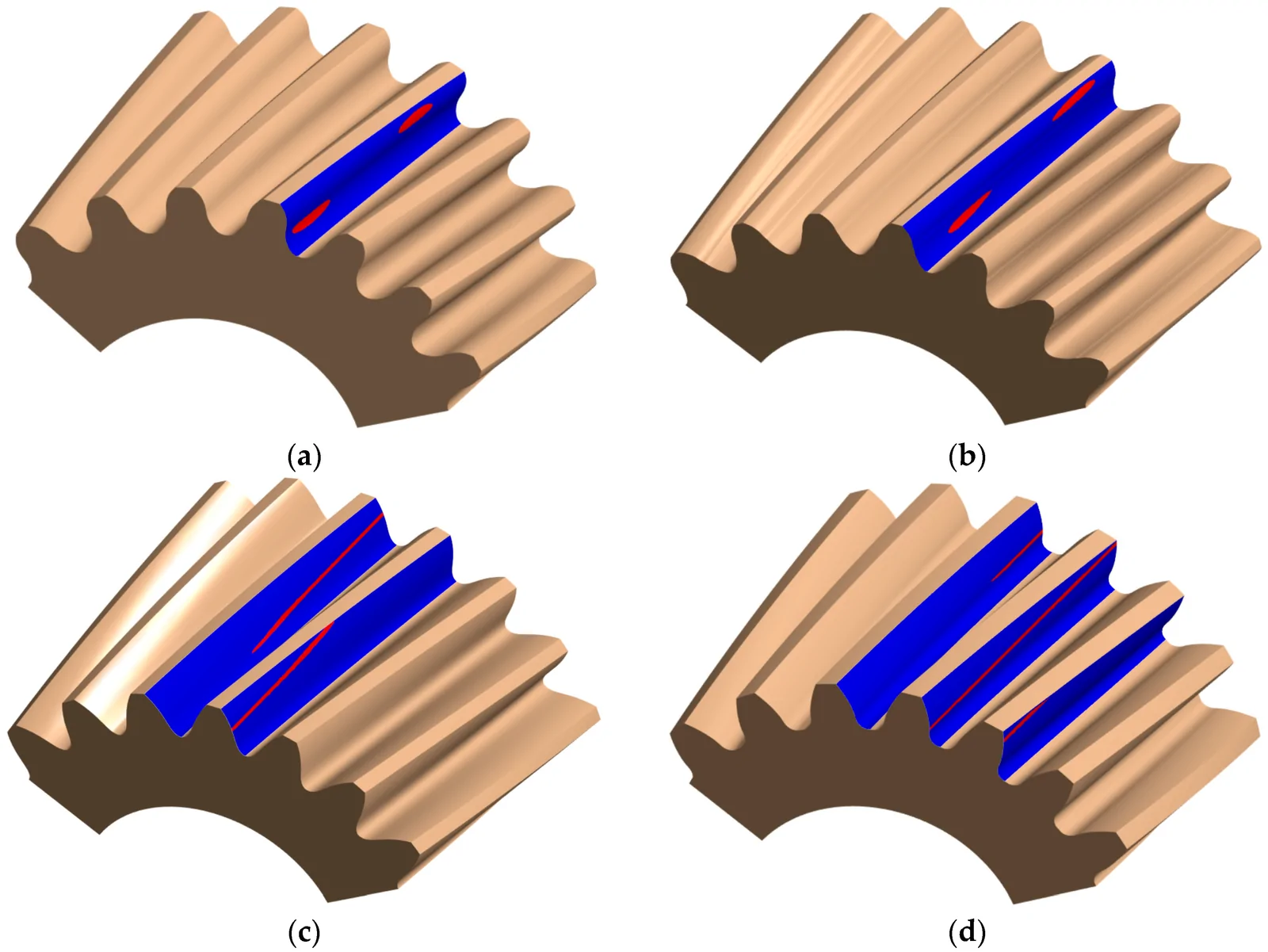
Instantaneous contact patterns for gears with the following profiles: (**a**) Novikov-69, (**b**) Novikov-96, (**c**) sinusoidal, (**d**) involute.

**Figure 14:**
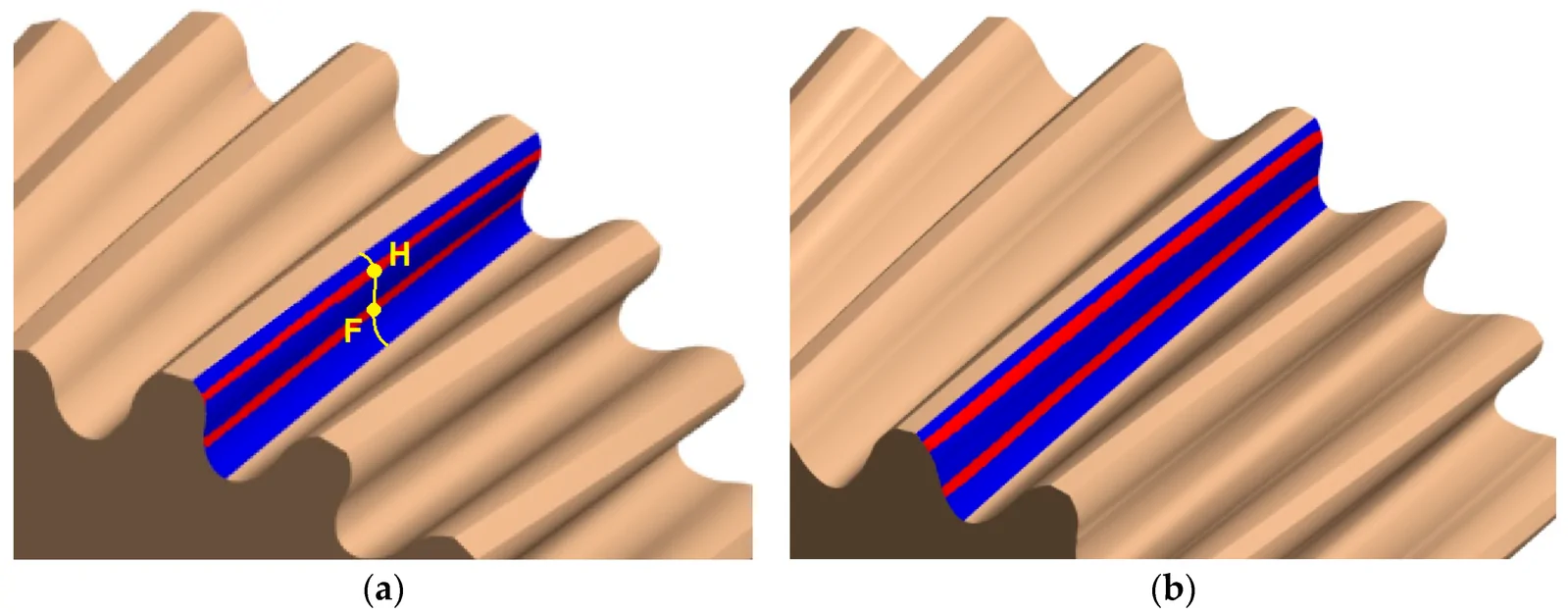

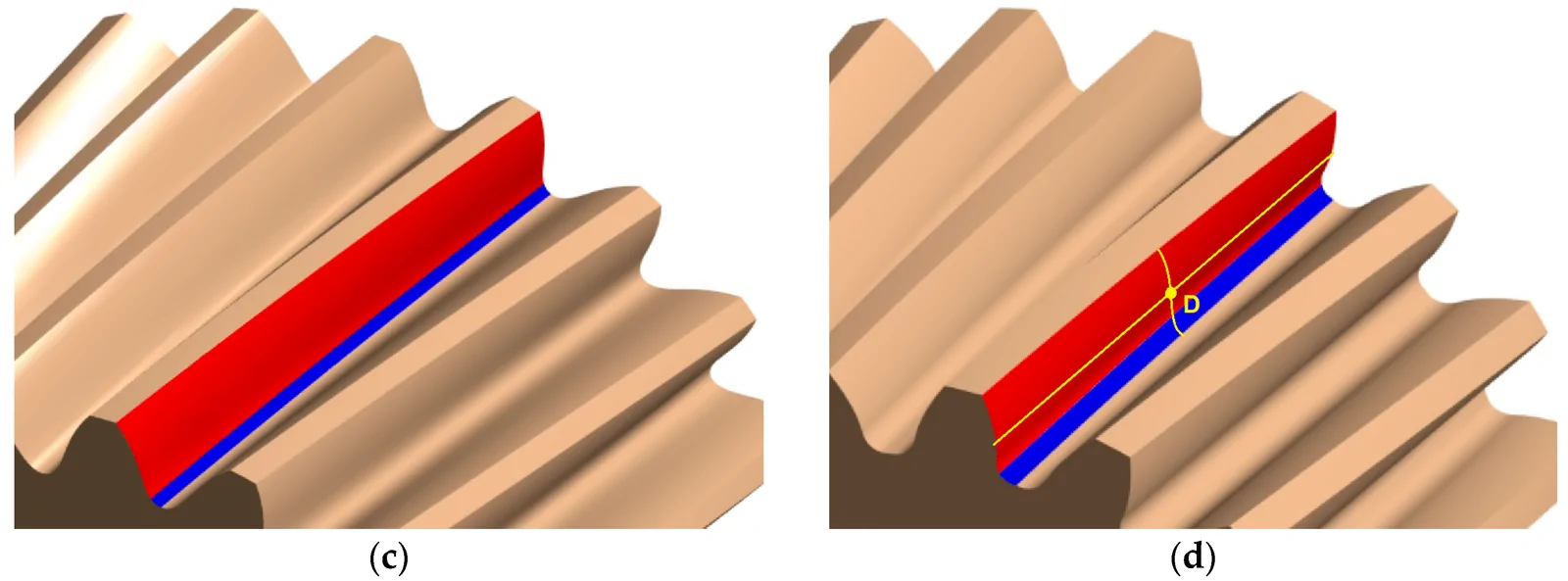
Total contact patterns for gears with the following profiles: (**a**) Novikov-69, (**b**) Novikov-96, (**c**) sinusoidal, (**d**) involute.

**Figure 15:**
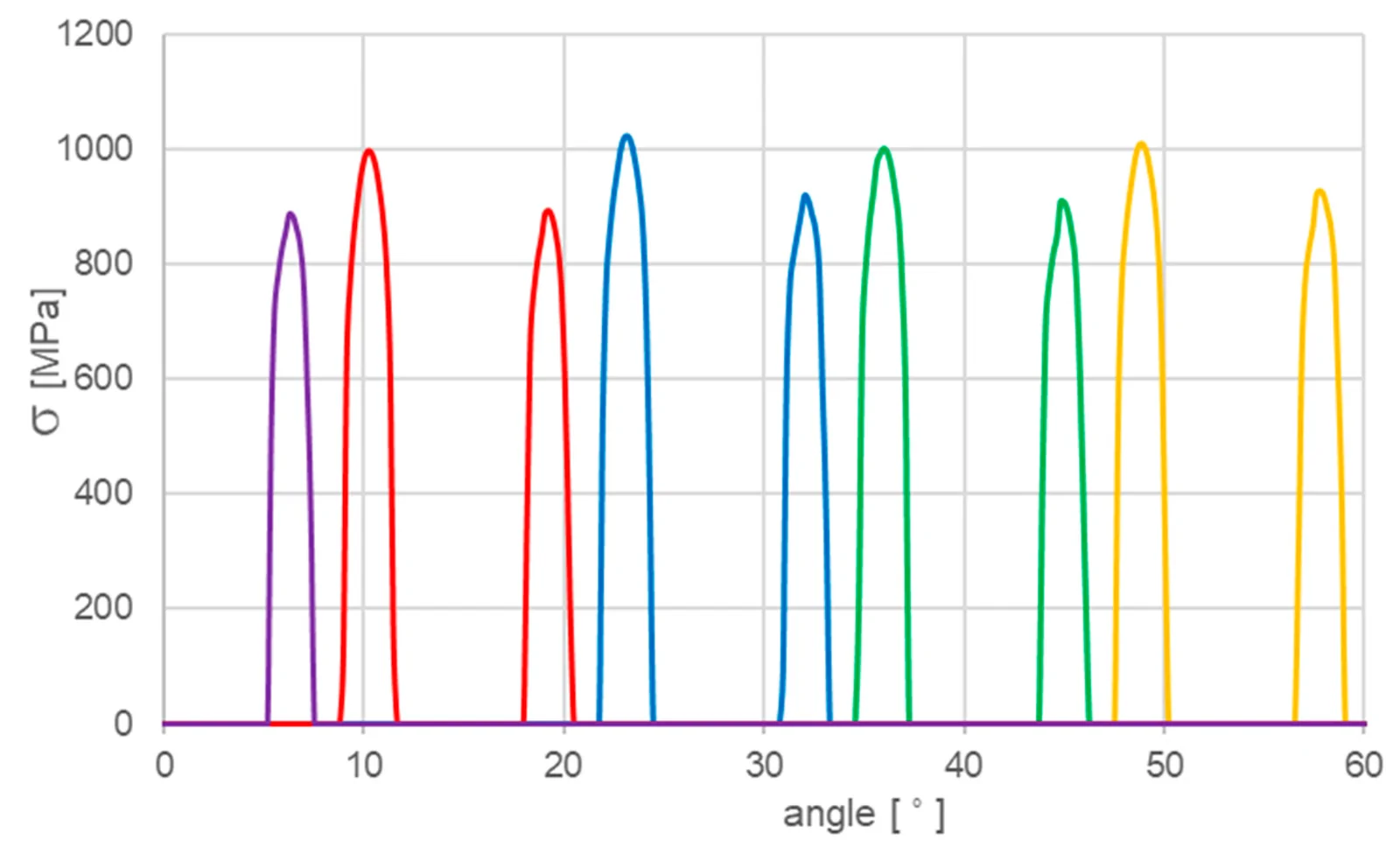
Plot of contact stresses at characteristic points on the contact lines for the Novikov-69 gear model as a function of the pinion rotation angle.

**Figure 16:**
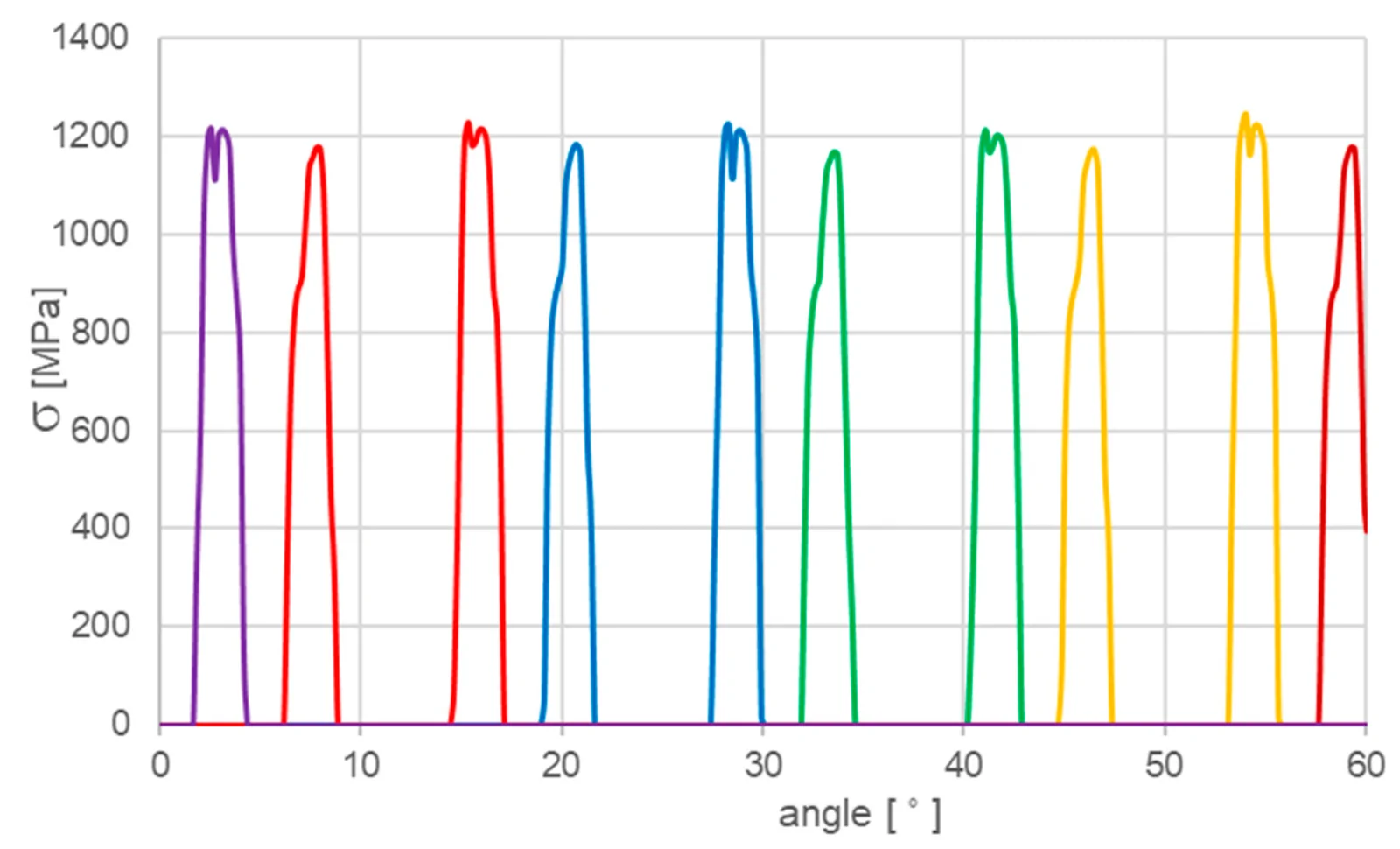
Plot of contact stresses at characteristic points on the contact lines for the Novikov-96 gear model as a function of the pinion rotation angle.

**Figure 17:**
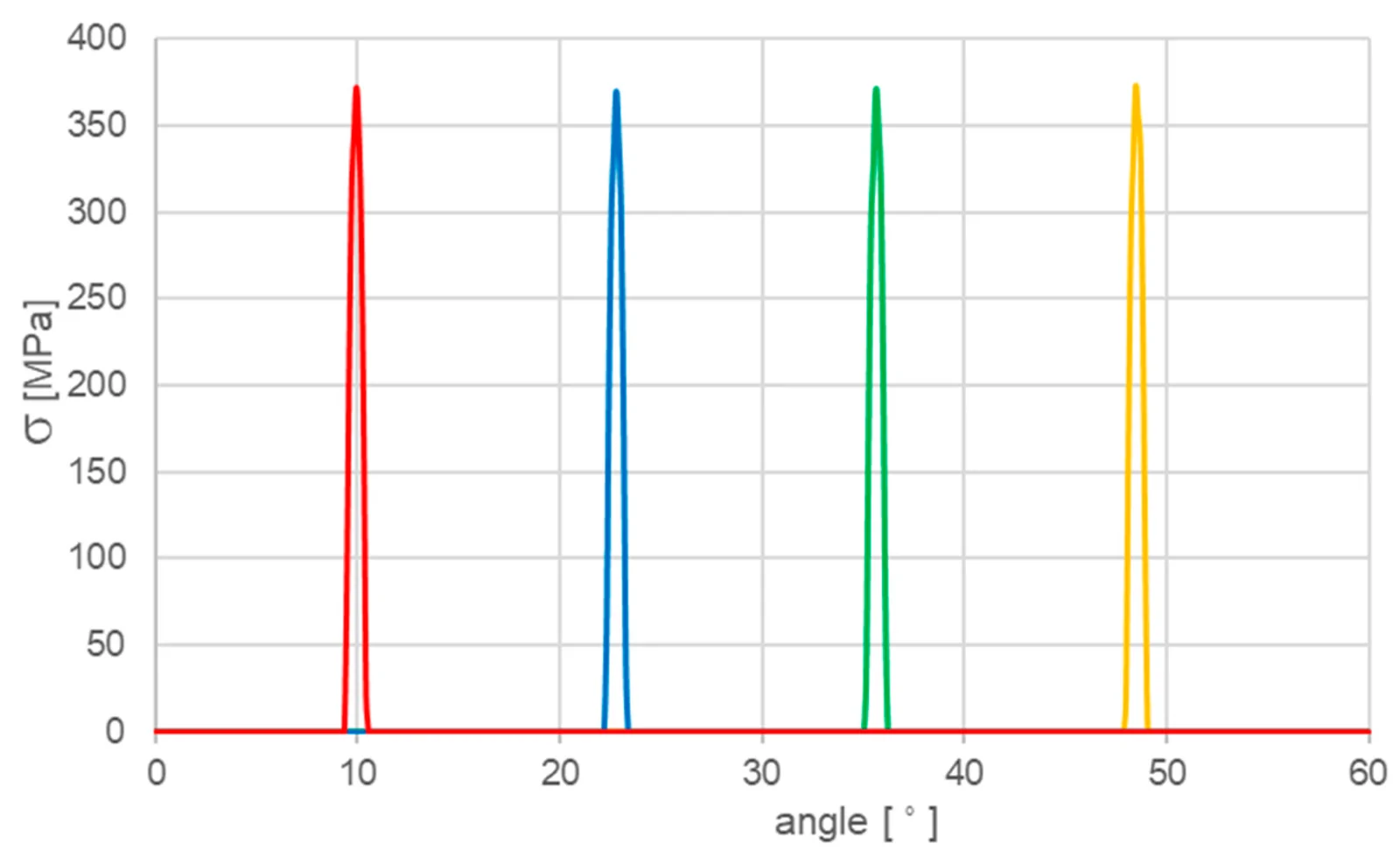
Plot of contact stresses at characteristic points on the contact lines for the sinusoidal gear model as a function of the pinion rotation angle.

**Figure 18:**
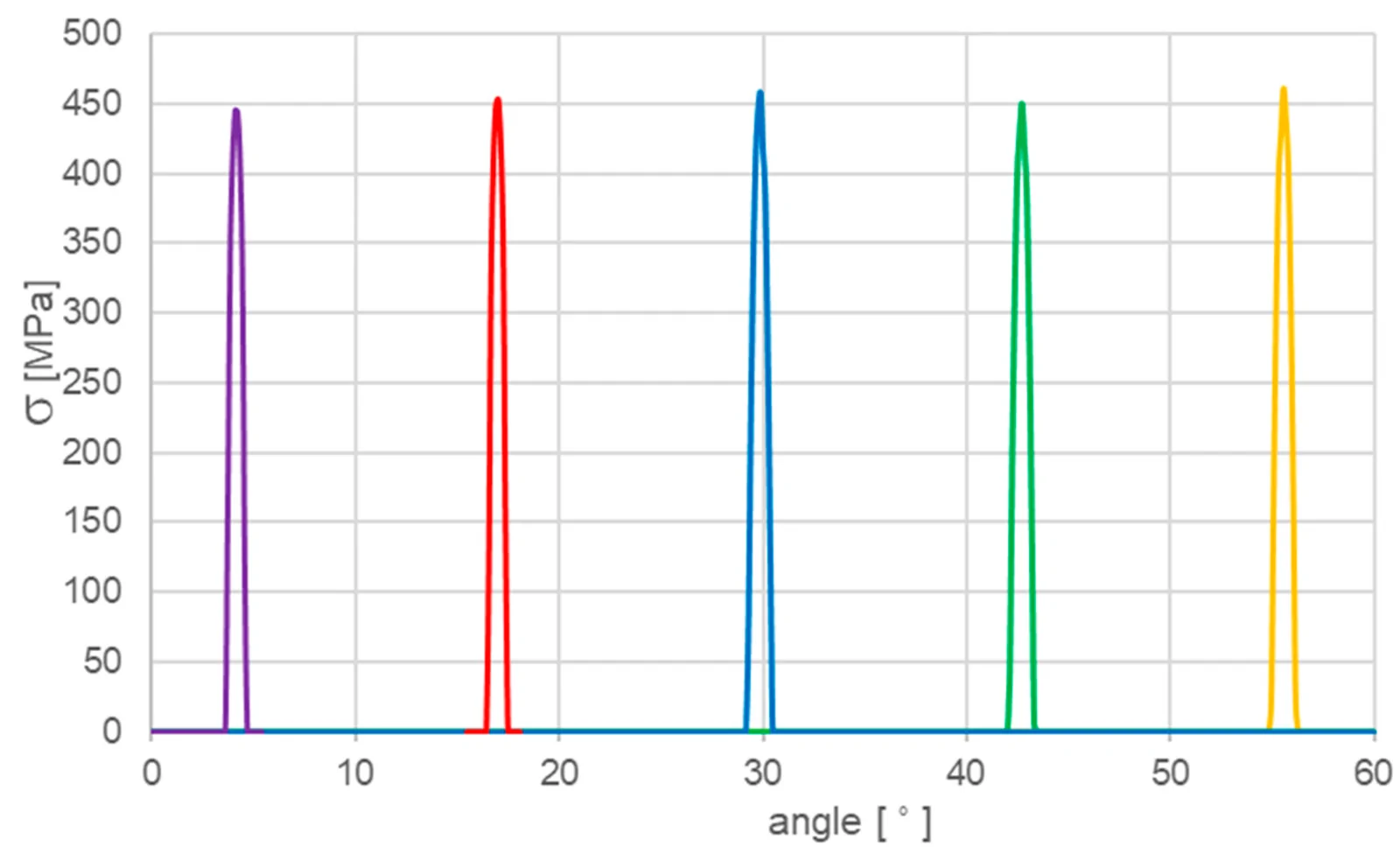
Plot of contact stresses at characteristic points on the contact lines for the involute gear model as a function of the pinion rotation angle.

**Table 1:** Parameter values of the basic rack profile for the Novikov-69 gear system with a normal module of m_n_ < 3.15 mm.

Parameter	GOST 15023-69 [mm]
ρ_a_	1.147
ρ_f_	1.307
ρ_p_	0.52246
l_a_	0.39270
l_f_	0.50526
h_a_	0.9
h_f_	1.05
h_k_	0.52073
S	1.53215
e	1.60944
c	0.15
α_k_	27°
j_k_	0.06

**Table 2:** Parameter values of the basic rack profile for the modified Novikov gear system for a normal module m_n_ in the range of 2.5 to 4.5 mm.

Parameter	GOST 30224-96 [mm]
ρ_a_	1.38
ρ_f_	1.76
ρ_i_	0.85
l_a_	0.64
l_f_	0.90128
h_a_	0.875
h_f_	1.01163
h_k_	0.59154
S	1.46797
e	1.67362
c	0.13663
α_k_	35°
j_k_	0.1

**Table 3:** Comparison of stress values in characteristic regions of the models for various tooth profiles.

Profile	Bending Stress [MPa]	Contact Stresses Maximum [MPa]	Contact Stresses Average [MPa]
Novikov-69	216.75	996.36	944.30
Novikov-96	170.70	1228.50	1202.90
sinosoidal	214.38	371.84	371.84
involute	118.50	453.52	453.52

## Data Availability

The original contributions presented in this study are included in the article. Further inquiries can be directed to the corresponding author.
